# The action of aminobenzacridines on the Furth rat leukaemia.

**DOI:** 10.1038/bjc.1969.102

**Published:** 1969-12

**Authors:** D. Shortridge, R. Turner, H. N. Green


### THE ACTION OF AMINOBENZACRIDINES ON THE FURTH RAT LEUKAEMIA

D. SHORTRIDGE, R. TURNER AND (THE LATE) H. N. GREEN

*From the Department of Experimental Pathology and Cancer Research, The School of Medicine, Leeds, LS2 9NL*

Received for publication July 18, 1969

HADDOW (1948) and Green (1954) showed that the carcinogenic angular polycyclic hydrocarbons were capable of both initiating and inhibiting the growth of some tumours. The purpose of this paper is to describe an attempt to modify the chemical structure of these compounds in such a way as to enhance their inhibitory effect.

It was felt that the problems of designing chemicals capable of a degree of differential destruction of tumour cells have much in common with those of leather technology. The dyeing of leather depends, in the first place, on charge differences between the dyestuff and the protein fibre to draw the reactants together and after this, firm bonding occurs due to the interplay of short range forces, such as dipole bonds, hydrogen bonds or van der Waals forces. Otto (1953), who was working with anionic dyestuffs, concluded that in the formation of bonds between aromatic substances and protein fibres the simplest kind of bond, which is the basis for all interactions, is an attraction between dipoles. On the other hand, in work on textile fibres, Derbyshire and Peters (1955) emphasised the importance of short range non-polar forces between the dye and the material being dyed.

Ambrose, James and Lowick (1956), amongst others, have shown that the tumour cell carries a greater degree of negative charge than normal cells of the same type. This suggested that a cancer chemotherapeutic agent based on the aromatic hydrocarbons would require a degree of positive charge (cationisation) to enable the agent to approach the negatively charged tumour surface more closely, and also a certain minimum planar area so that it would bond adequately with the tumour cell receptors through short range forces. Such bonding requires the molecules of the agent and the cell surface to come into close proximity and implies a precise complementary shape for the reactants.

The relationship of chemical structure to carcinogenic properties of the polycyclic hydrocarbons and benzacridines is well known (Badger, 1948; Laccassagne, Buu-Hoï, Daudel and Zajdela, 1956; Clayson, 1962). Pullman and Pullman (1955) believed that the effect of substituents is to alter the overall electronic pattern in the polycyclic hydrocarbons, and Laccassagne *et al.* (1956) were of the same opinion in respect of the benzacridines.

The work of Albert, Goldacre and Phillips (1948) on the connection between chemical structure and basic strength in nitrogen substituted aromatic bases and the description of the relationship between antibacterial potency and the summation of multiple weak bonds in the acridine series (Albert, Ribbo and Burvill, 1949) led us to choose the aminobenzacridines for this investigation.

The numbering of substituted benzacridines is in a state of considerable confusion. In this paper we have adopted the system of ring numbering used in *Chemical Abstracts* as shown in Fig. 1.

FIG. 1.

### MATERIALS AND METHODS

### *Biological test system*

The Furth rat leukaemia and the Chester Beatty Research Institute-derived Wistar rats have been described previously (Green and Shortridge, 1967). In these experiments all rats inoculated with blood containing 750,000 white cells from leukaemic animals died, without further treatment, from 9–16 days later with naked eye evidence of leukaemia at post mortem. When a group of these animals received an inoculation from the same sample of blood they almost invariably died within 2 days of each other.

### *Preparation of solutions*

The chemicals were dissolved or suspended in one or more of the following vehicles:

1. 70% propylene glycol, 0·025M phosphate buffer pH 7·4. (Experience showed this to be the most suitable for many compounds.)
2. Dry ground in normal saline.
3. 70% propylene glycol, 0·025M phosphate buffer pH 7·0.
4. 20% propylene glycol, pH 6·8.
5. Normal saline, pH 5·5.
6. 20% propylene glycol, pH 7·1.
7. Normal saline, pH 5·0.

*Procedure*

Groups of four or five compounds, including 7-aminobenz(c)acridine, were set up with each group of control rats; 6–10 rats were used for each compound.

Except where it is stated otherwise, solutions were injected s.c. at a different site on each occasion, beginning between the first and the fourth day following the inoculation of the tumour. The chemicals were usually given daily up to 7 days after inoculation. Thereafter, chemical treatment was continued in groups of animals with high white cell counts in the peripheral blood but was withheld from groups in which the counts were near normal. The magnitude of the dose was determined by the amount of chemical available and its toxicity.

Many of the administered chemicals led to a s.c. reaction with local inflammation and oedema, followed in most cases by some ulceration of the skin.

*Chemicals*

All the benzacridine derivatives and related compounds were prepared in the laboratory. 7-Aminobenz(c)acridine, 12-aminobenz(a)acridine, 9-aminobenz(c)-acridine, 4-aminobenz(h)quinoline (Albert, Brown and Duewell, 1948), 10-amino-benz(c)acridine (Albert, 1948), 7-N-(2′-hydroxyethyl)aminobenz(c)acridine (Elslager, Moore, Short, Sullivan and Tendick, 1957), 7-(3′-diethylaminopropylamino)-benz(c)acridine hydrochloride, 7-chlorobenz(c)acridine (Bachman and Picha, 1946), 7-phenoxybenz(c)acridine (Moore, Elslager and Short, 1961) and 7-aminobenz(b)-(1,10)phenanthroline (Wilkinson and Finar, 1948) had the same characteristics as described in the previous literature.

New compounds were prepared (Table I) by various methods:

A. 7-(*N-Alkylamino*)-*benz*(*c*)*acridines*. To 7-phenoxybenz(c)acridine (0·01 mole) in phenol (20 g.) at 70° C. was added the appropriate amine hydrochloride (0·01 mole) and the mixture heated to 120° C. for 2 hours. The cooled solution was poured into ether and stood at 4° C. overnight. The solid was dissolved in methanol and reprecipitated with ether. It was extracted into water and the product precipitated with alkali and crystallised from benzene-petroleum spirit.

B. 9-*Methoxy*-7-*N*-(2′-*hydroxyethyl*)*aminobenz*(*c*)*acridine*. 7-Chloro-9-methoxybenz(c)acridine (Bachman and Picha, 1946) (5 g.) and ethanolamine (25 ml.) were heated for 4 hours at 120° C. The cooled mixture was added to acetone (200 ml.) and kept at 4° C. overnight. Unreacted starting material was removed by filtration and the product precipitated by the addition of 10N HCl (50 ml.). Crystallisation was induced by trituration with isopropanol. The residue was extracted with water and the free base thrown out with 2N NaOH. Crystallisation from ethanol gave the desired material (3·5 g., 65%).

C. 7-*Amino*-10-*nitrobenz*(*c*)*acridine*. 2-Chloro-4-nitrobenzoic acid and 1-naphthylamine were reacted by the method of Lésnianski (1929) to give N-(1′-naphthyl)-4-nitroanthranilic acid (m.p. >300° C., 46%). This with phosphoryl chloride gave 7-chloro-10-nitrobenz(c)acridine (m.p. >300° C., 73%). To the latter (0·01 mole) in phenol (40 g.) at 120° C. was added powdered ammonium carbonate (0·015 mole) over 10 minutes followed by further heating for 1 hour. The product was precipitated by addition of the mixture to excess 2N NaOH and finally crystallised from nitrobenzene.

7-*Amino*-10-*chlorobenz*(*c*)*acridine* was similarly prepared from 7,10-dichlorobenz(c)acridine (Elslager *et al.*, 1957).

D. 7,9-*Diaminobenz*(*c*)*acridine*. N-(1′-Naphthyl)-5-nitroanthranilic acid (Lésnianski, 1929) (0·094 mole, 29 g.) was refluxed with phosphoryl chloride (500 ml.) for 2 hours. After the excess phosphoryl chloride had been distilled, the residue was thinned with chloroform (200 ml.) and added to a large excess of ammonia and ice to obtain 7-chloro-9-nitrobenz(c)acridine (m.p. 252–254° C., 81%). This was converted into 7-amino-9-nitrobenz(c)acridine by method C (m.p. >300° C., 97%). The latter (0·013 mole, 3·8 g.) was added portion-wise to a stirred boiling solution of stannous chloride (0·055 mole) in 10N HCl (45 ml.). Further 10N HCl was added (20 ml.). The solution was stirred at 100° C. for 2 hours, cooled and 30% NaOH (100 ml.) added. The precipitate was extracted with hot 10% acetic acid (2 × 100 ml.) and a trace of sodium hydroxide solution added to throw out black solid matter. Treatment of the filtered solution with excess sodium hydroxide gave 7,9-diaminobenz(c)acridine which was crystallised from benzene (m.p. 163–164° C., 53%).

E. 7-*Acetamidobenz*(*c*)*acridine*. The amine (0·01 mole) was heated with acetic anhydride (12 ml.) for 0·5 hour at 100–110° C. The solid was washed with benzene and hot alcohol (m.p. >300° C., 81%).

F. 7-(*N-Alkylamino*)-9-*methoxybenz*(*c*)*acridines*. These were made by method A using 7-phenoxy-9-methoxybenz(c)acridine (obtained from 7-chloro-9-methoxybenz(c)acridine by reaction with phenol) as starting material.

G. 5-*Ethyl*-7-*aminobenz*(*c*)*acridine*. N-(4′-Ethyl-1′-naphthyl)anthranilic acid, m.p. 177–9° C., was obtained from 4-ethyl-1-naphthylamine and 2-chlorobenzoic acid by the Jourdan-Ullmann reaction (Bachman and Picha, 1946), yield 32%. This was converted to 5-ethyl-7-chlorobenz(c)acridine, m.p. 125–127° C. with phosphoryl chloride, yield 33%. The amination was then performed by method C.

H. 7-(2′-*Propanediol*)*aminobenz*(*c*)*acridine*. 7-Chlorobenz(c)acridine (0·02 mole) was dissolved in phenol (40 g.) at 70° C. 1-Amino-2,3-propanediol (0·034 mole) was added and the mixture heated at 120° C. for 3 hours. The cooled solution was poured into acetone and the resulting solid dissolved in methanol and reprecipitated with ether. An aqueous solution of this was made alkaline and the thick oil obtained, crystallised from ethanol.

Chemicals were characterised by their melting point and elementary analysis (Table I). U.V. spectra were determined on a Unicam S.P. 700 Recording Spectrophotometer and I.R. spectra on a Perkin Elmer 257 Spectrophotometer. pKa values were determined by the potentiometric titration method of Albert and Serjeant (1962) using 0·0005 g. mole of the chemical in the solvents indicated in Table I. These were corrected to the values to be expected in aqueous solution by the method of Albert *et al.* (1949).

### RESULTS AND COMMENT

The first series of experiments (Table II) demonstrated that 7-aminobenz(c)-acridine was consistently and significantly effective in prolonging the life of rats carrying the Furth leukaemia if it were administered on several occasions by s.c. injection. Single injections or the compound in the diet were ineffective.

It appears that with these compounds four conditions must be satisfied at the same time for anti-tumour activity: (i) the charge on the molecule, which is

TABLE I.—*Analysis and Properties of New Benzacridines Described in this Work*

| Substituent on 7-amino group | Other substituents | m.p. ° C. | Calculated (%) C | Calculated (%) H | Calculated (%) N | Found (%) C | Found (%) H | Found (%) N | pKa | pKa (corr.) | % Yield | Method |
|---|---|---|---|---|---|---|---|---|---|---|---|---|
| —$CH_3$ | — | 110–111 | 83·7 | 5·4 | 10·9 | 84·1 | 5·4 | 10·9 | 8·2a | 8·9 | 62 | A |
| —$CH_2CH_3$ | — | 95–96 | 83·8 | 5·9 | 10·3 | 83·9 | 6·1 | 10·5 | 7·9a | 8·6 | 78 | A |
| —$CH_2CH_2CH_3$ | — | 106–107 | 83·9 | 6·3 | 9·8 | 84·0 | 6·3 | 9·8 | 7·9a | 8·6 | 75 | A |
| —$CH(CH_3)_2$ | — | 142–143 | 83·9 | 6·3 | 9·8 | 84·1 | 6·5 | 10·0 | 7·4a | 8·1 | 49 | A |
| —$CH_2CH_2CH_2CH_3$ | — | 76 | 84·0 | 6·7 | 9·3 | 84·0 | 6·9 | 8·9 | 7·7a | 8·4 | 50 | A |
| —CH($CH_2CH_3$)$CH_3$ | — | 108–109 | 84·0 | 6·7 | 9·3 | 84·1 | 6·9 | 9·5 | 7·6a | 8·3 | 43 | A |
| —$CH_2CH(CH_3)_2$ | — | 96–97 | 84·0 | 6·7 | 9·3 | 84·2 | 7·0 | 8·9 | 7·7a | 8·4 | 41 | A |
| —$CH_2CH_2OH$ | 9-$OCH_3$ | 155–157 | 75·5 | 5·7 | 8·8 | 75·3 | 5·8 | 8·7 | 7·2a | 7·9 | 65 | B |
| —H | 10-Cl | 240–241 | 73·2 | 4·0 | 10·1 | 73·0 | 4·0 | 10·1 | 7·2b | 7·9 | 82 | C |
| —H | 10-$NO_2$ | 230 | 70·6 | 3·9 | 14·5 | 70·0 | 3·8 | 14·1 | 7·3c | 8·0 | 46 | C |
| —H | 9-$NH_2$ | 163–164 | 78·9 | 5·0 | 16·2 | 79·0 | 5·2 | 16·2 | 8·3a | 9·0 | 53 | D |
| —$COCH_3$ | — | >300 | 79·7 | 4·9 | 9·8 | 79·5 | 5·0 | 10·0 | — | — | 81 | E |
| —$CH_2CH_3$ | 9-$OCH_3$ | 122–123 | 79·5 | 6·0 | 9·3 | 79·3 | 6·2 | 9·2 | 7·6a | 8·3 | 79 | F |
| —$CH(CH_3)_2$ | 9-$OCH_3$ | 134–136 | 79·8 | 6·3 | 8·9 | 79·4 | 6·4 | 9·2 | 7·0a | 7·7 | 49 | F |
| —H | 5-$C_2H_5$ | 182–185 | 83·8 | 5·9 | 10·3 | 84·2 | 5·9 | 10·1 | 8·3a | 9·0 | 61 | G |
| —$CH_2CHOHCH_2OH$ | — | 132–135 | 75·5 | 5·7 | 8·8 | 75·9 | 5·9 | 8·7 | 7·6a | 8·3 | 66 | H |

Solvents used for pKa determinations:

a—ethanol : water (1 : 1).

b—ethanol : water (2 : 1).

c—propylene glycol : water (1 : 1).

TABLE II.—*Variation in the Method of Administration of 7-Aminobenz(c)-acridine and Activity Against the Furth Rat Leukaemia*

| Method of administration | Frequency | pH of medium | % Increase in survival over that of controls in separate experiments | Dose of chemical (mg.) |
|---|---|---|---|---|
| s.c. injection | multiple | ≥7 | 97, 68, 45, 43<br>43, 63, 77, 55 | 35–65 |
| s.c. injection | single | ≥7 | 20, 35 | 15–25 |
| s.c. injection | multiple | <7<br>≤5 | 65, 42, 72<br>0, 12 | 40–70<br>35–100 |
| 1 % in diet | daily | — | 25 | — |

dependent on the pKa value; (ii) the distribution of this charge within the molecule; (iii) the shape of the molecule which determines its fit on the receptor; and (iv) the surface area of the molecule which determines the stability of combination with the receptor. Thus, adequate ionisation is needed but is not sufficient in itself because, although 7-aminobenz(c)acridine is active, the similarly ionised 12-aminobenz(a)acridine and 4-aminobenz(h)quinoline are without activity (Table III). The critical importance of charge distribution is shown by comparing the two former compounds. Similarly the shape of the molecule by itself is inadequate as 7-chlorobenz(c)acridine has approximately the same spatial characteristics as 7-aminobenz(c)acridine but is devoid of activity. Finally the area of the molecule must exceed that of 4-aminobenz(h)quinoline or 9-aminoacridine.

Attempts to enhance the activity of 7-aminobenz(c)acridine have proved unsuccessful. Only the 7-N-methyl and 7-N-ethyl derivatives have shown slight and variable activity against the Furth leukaemia. Substitution of the 7-amino group with *n*-propyl, *n*-butyl, 3′-diethylaminopropyl, 2′-hydroxyethyl or phenyl led to inactive compounds. Similarly, ring substitution, as in 7,9- and 7,10-diaminobenz(c)acridine, 7-amino-9-nitrobenz(c)acridine and 7-amino-10-chlorobenz(c)acridine, led to inactive compounds. The aza substituted derivative, 7-aminobenz(b)(1,10)-phenathroline, and 7-phenoxybenz(c)acridine were also without significant activity.

The acute toxicity of 7-aminobenz(c)acridine was assessed after subcutaneous injection as LD50 = 22 mg./100 g. body weight. Administration of 6 daily doses of 5 mg./100 g. body weight to 6 rats led to a loss in weight of 15% and reduction in the total white cell count to 26% of the normal value. The white cell count began to rise 4–5 days after the first dose and showed a marked neutrophilia. Lymphocytes recovered more slowly. Further investigations should be directed to preparing aminobenzacridines with a higher therapeutic ratio. Some of these chemicals have been forwarded to European Organisation for Research on Treatment of Cancer (GECA) for fuller investigation.

### SUMMARY

1. Reasons have been advanced for the study of aminobenzacridines as cancer therapeutic agents.

2. Of 20 compounds tested only one, 7-aminobenz(c)acridine, showed an inhibitory effect on the test tumour, the Furth rat leukaemia.

We thank G. Littlewood, A.I.M.L.T., for the maintenance and treatment of the animals and Professor D. B. Clayson for advice in the preparation of the manu-

TABLE III.—*Correlation Between Structure, pKa and Activity Against the Furth Rat Leukaemia*

| Compound | pKa (corr.) | % ionised pH 7·3 | 1st dose (days post inoculation) | Daily dose (mg.) | Total dose (mg.) | Vehicle* | No. of rats treated/ controls | Surviva (days) treated/ controls | % increase |
|---|---|---|---|---|---|---|---|---|---|
| 7-Aminobenz(c)acridine | 8·8 | 95 | 4 | 5 | 35 | 1 | 6/6 | 28·0/14·3 | 97 |
| 12-Aminobenz(a)acridine | 9·1 | 98 | 4 | 5 | 20 | 1 | 6/6 | 15·0/14·3 | 5 |
| 9-Aminobenz(c)acridine | 4·7 | 0·25 | 4 | 2, 4, 5 | 16 | 1 | 6/6 | 14·3/14·3 | 0 |
| 10-Aminobenz(c)acridine | 6·7 | 20 | 3 | 4 | 16 | 2 | 6/8 | 8·5/9·1 | 0 |
| 4-Aminobenzo(h)quinoline | 8·6 | 95 | 4 | 5, 10 | 25 | 1 | 6/6 | 12·5/11·6 | 9 |
| 9-Aminoacridine hydrochloride | 9·6 | 99 | 4 | 5, 2 | 12 | 1 | 6/6 | 11·6/11·6 | 0 |
| 7-Chlorobenz(c)acridine | — | — | 1 | 2, 4, 5, 7·5 | 39 | 2 | 6/9 | 11·8/12·0 | 0 |

* See text.

script. The Yorkshire Council of the British Empire Cancer Campaign for Research supported this investigation.

### REFERENCES

ALBERT, A.—(1948) *J. chem. Soc.*, p. 1225.
ALBERT, A., BROWN, D. J. AND DUEWELL, H.—(1948) *J. chem. Soc.*, p. 1284.
ALBERT, A., GOLDACRE, R. AND PHILLIPS, J. L.—(1948) *J. chem. Soc.*, p. 2240.
ALBERT, A., RIBBO, S. D. AND BURVILL, M. I.—(1949) *Br. J. exp. Path.*, **30**, 159.
ALBERT, A. AND SERJEANT, E. P.—(1962) ' Ionization Constants of Acids and Bases '. London (Methuen and Co.).
AMBROSE, E. J., JAMES, A. M. AND LOWICK, J. H. B.—(1956) *Nature, Lond.*, **177**, 576.
BACHMAN, G. B. AND PICHA, G. M.—(1946) *J. Am. chem. Soc.*, **68**, 1599.
BADGER, G. M.—(1948) *Br. J. Cancer*, **2**, 309.
CLAYSON, D. B.—(1962) ' Chemical Carcinogenesis '. London (Churchill).
DERBYSHIRE, A. N. AND PETERS, R. H.—(1955) *J. Soc. Dyers Colour.*, **71**, 530.
ELSLAGER, E. F., MOORE, A. M., SHORT, F. W., SULLIVAN, M. J. AND TENDICK, F. H.—(1957) *J. Am. chem. Soc.*, **79**, 4703.
GREEN, H. N.—(1954) *Br. med. J.*, ii, 1374.
GREEN, H. N. AND SHORTRIDGE, D.—(1967) *Nature, Lond.*, **215**, 71.
HADDOW, A.—(1948) *J. Path. Bact.*, **47**, 567.
LACCASSAGNE, BUU-HOÏ, N. P., DAUDEL, R. AND ZAJDELA, P.—(1956) *Adv. Cancer Res.*, **4**, 315.
LÉSNIANSKI, W.—(1929) *Bull. int. Acad. pol. Sci. Lett.*, Série A, p. 81.
MOORE, A. M., ELSLAGER, E. F. AND SHORT, F. W.—(1961) *U.S. Pat. Q.*, **2**, 981, 731.
OTTO, G.—(1953) *Leder*, **4**, 193.
PECK, R. M., PRESTON, R. K. AND CREECH, H. J.—(1959) *J. Am. chem. Soc.*, **81**, 3984.
PULLMAN, A. AND PULLMAN, B.—(1955) *Adv. Cancer Res.*, **3**, 117.
WILKINSON, J. H. AND FINAR, I. L.—(1948) *J. chem. Soc.*, p. 288.


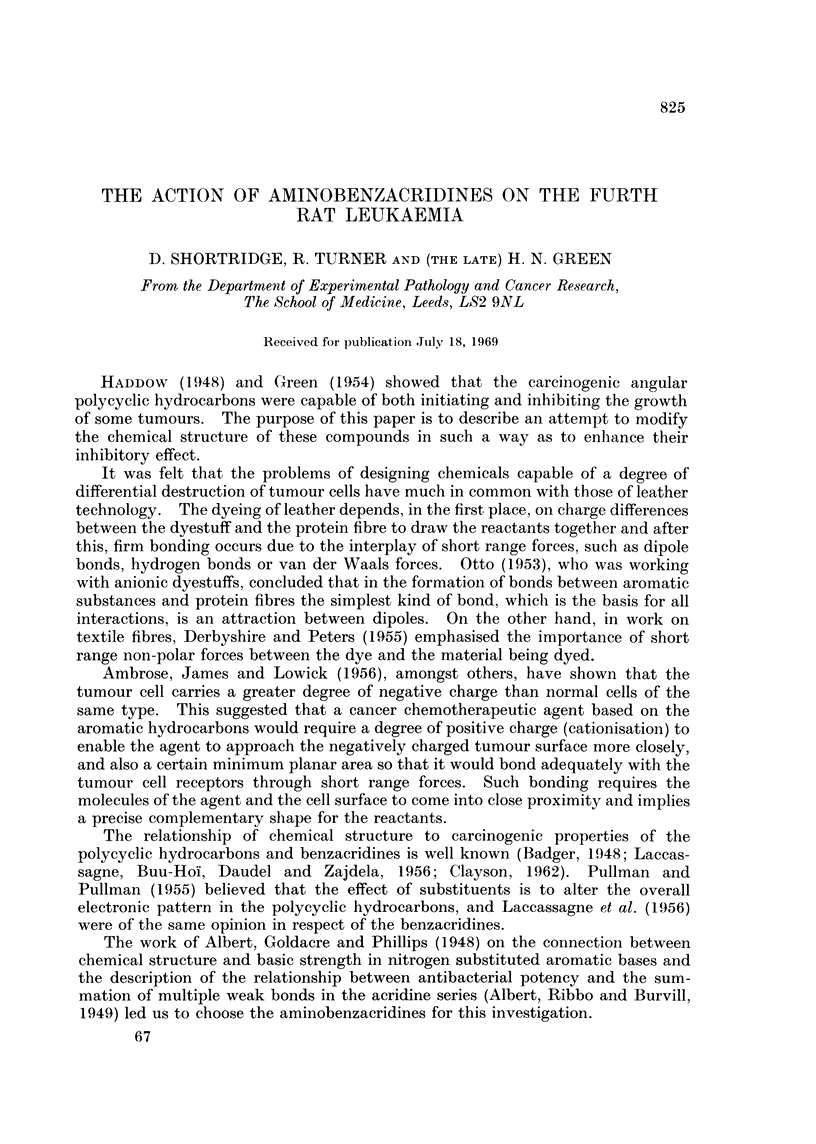

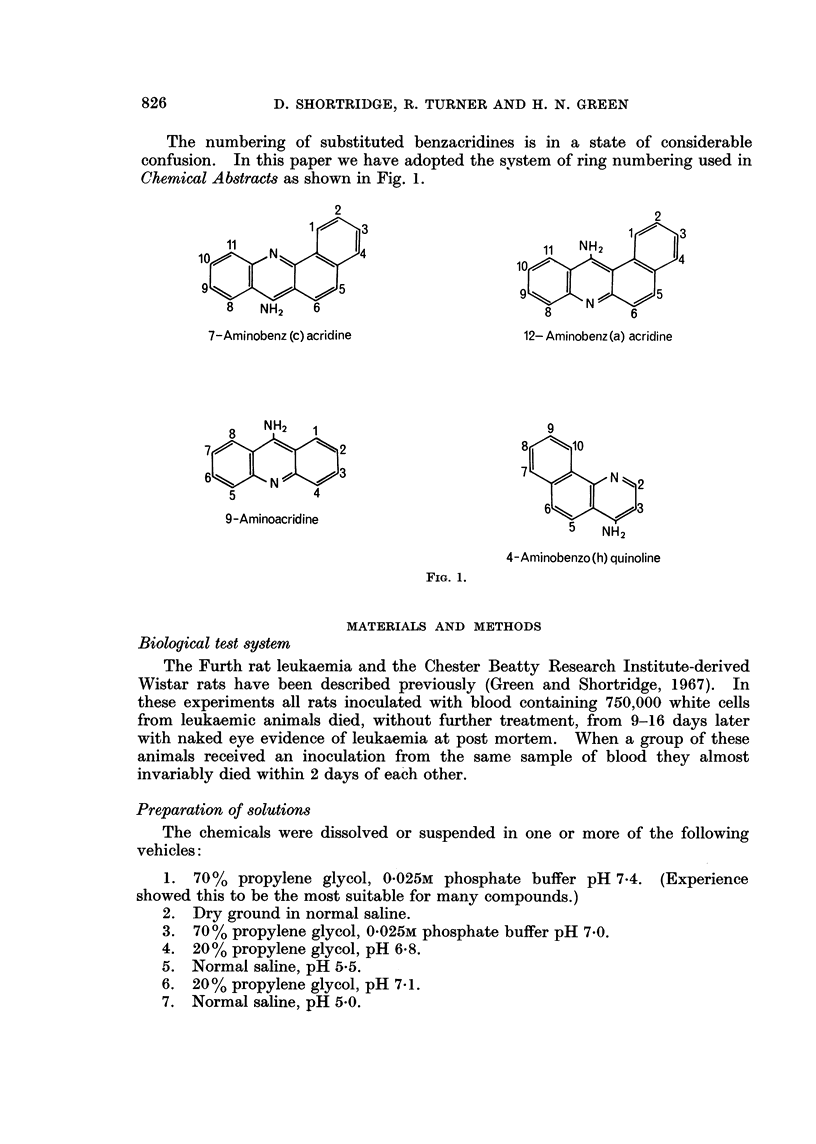

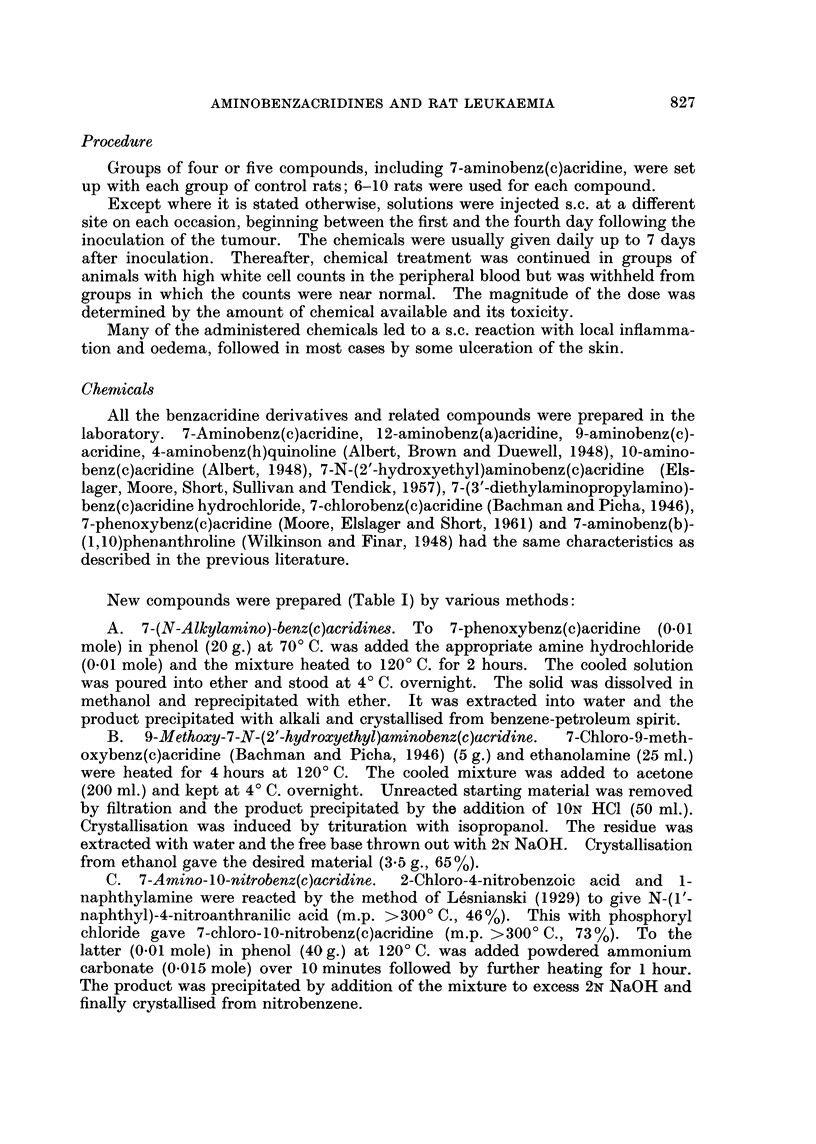

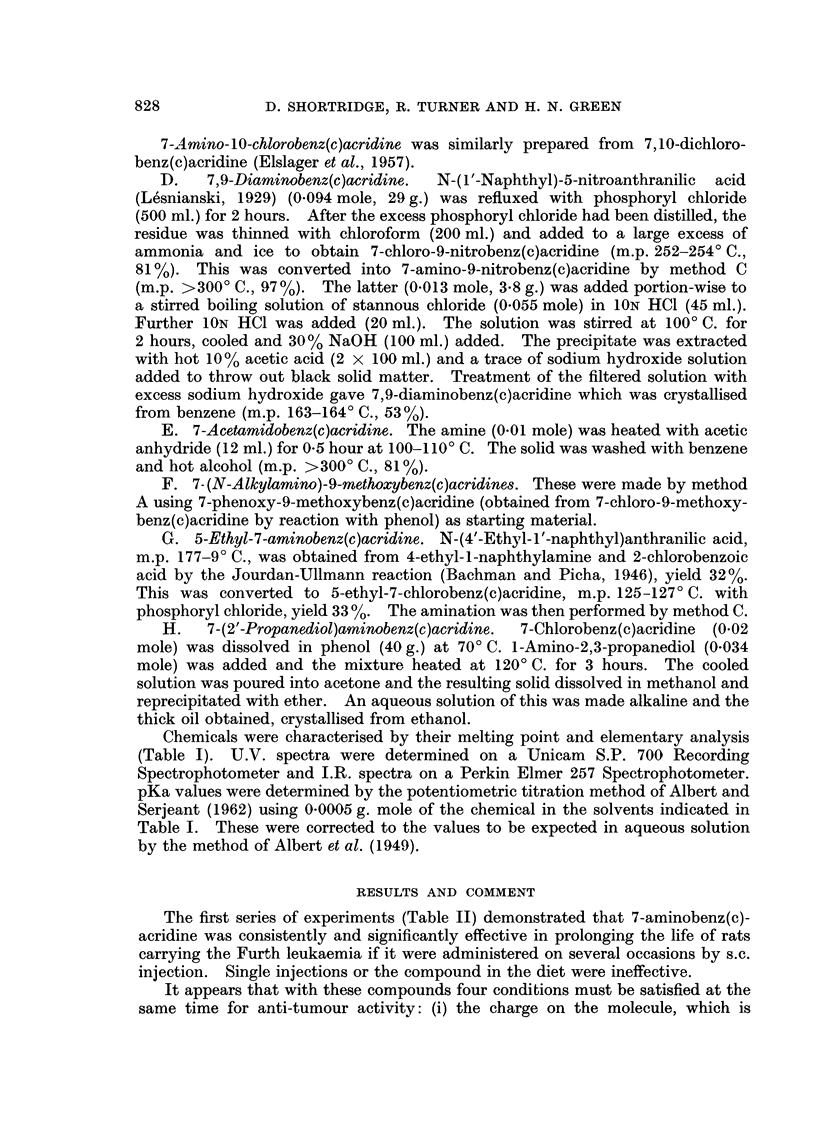

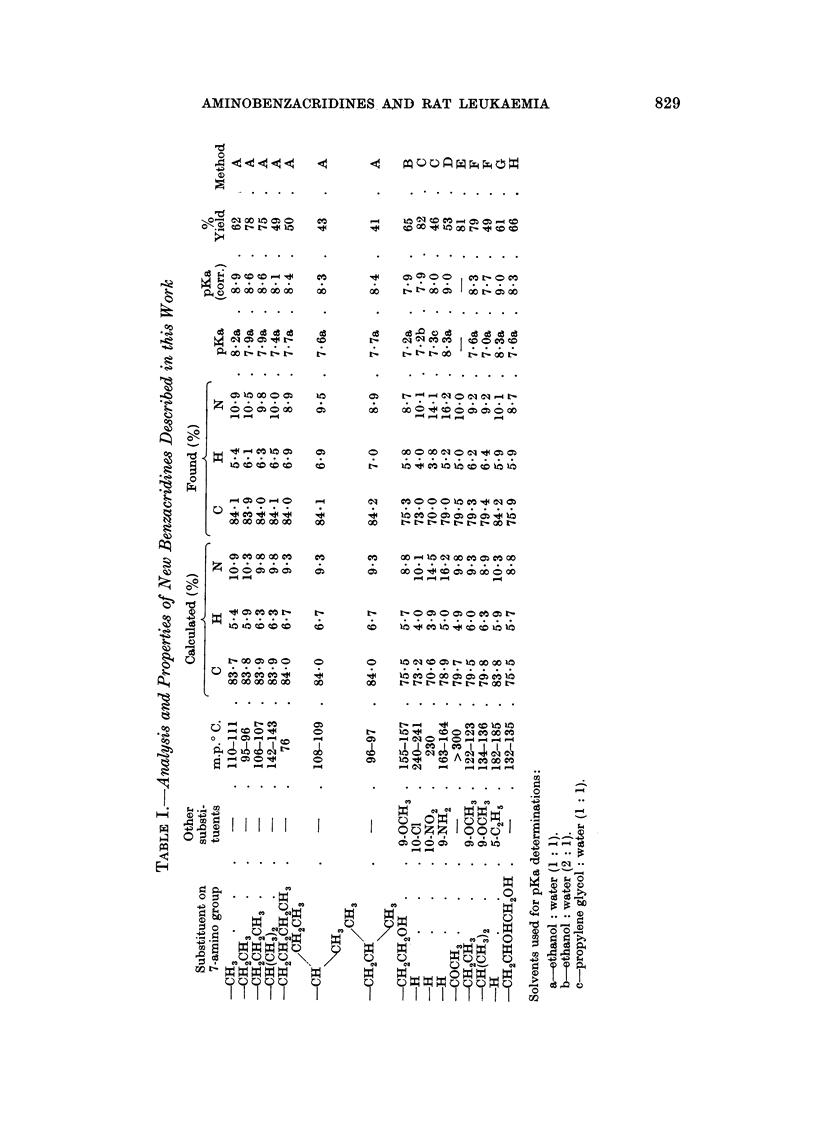

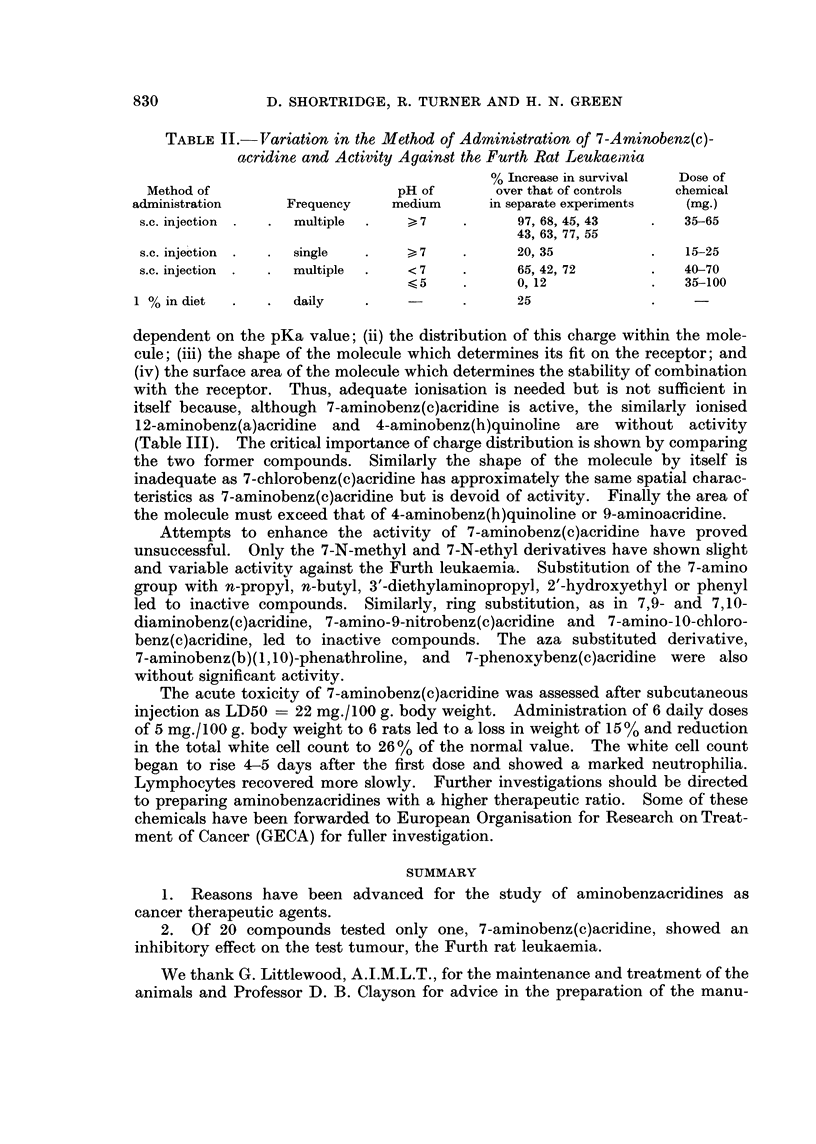

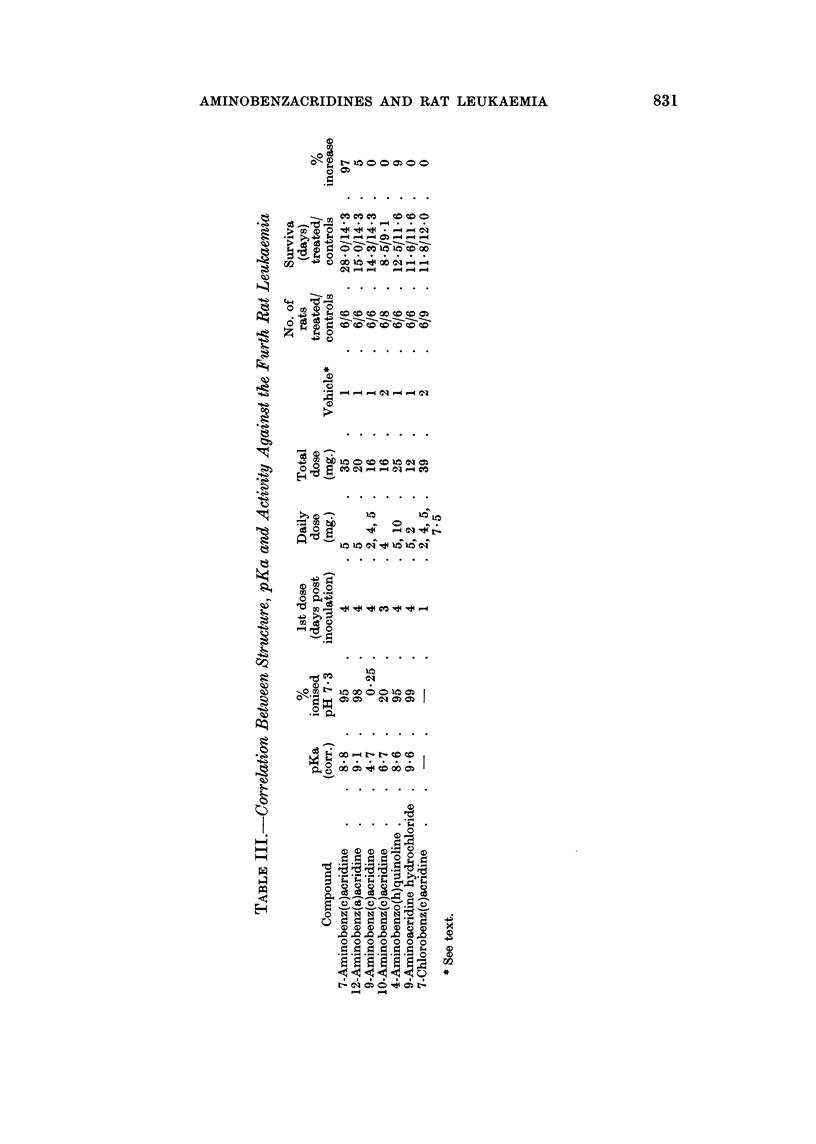

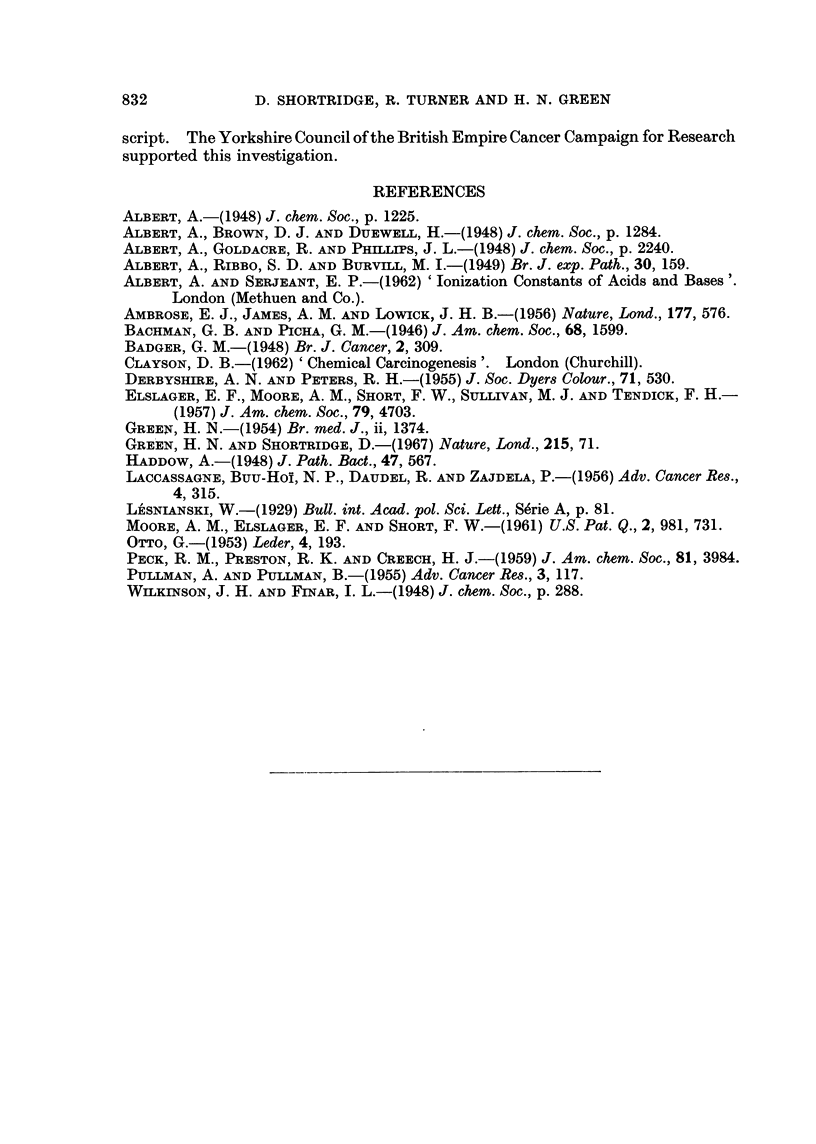


## References

[OCR_00525] BUU-HOI N. P., DAUDEL R., LACASSAGNE A., ZAJDELA F. (1956). The relation between carcinogenic activity and the physical and chemical properties of angular benzacridines.. Adv Cancer Res.

[OCR_00509] JAMES A. M., AMBROSE E. J., LOWICK J. H. (1956). Differences between the electrical charge carried by normal and homologous tumour cells.. Nature.

[OCR_00535] PULLMAN A., PULLMAN B. (1955). Electronic structure and carcinogenic activity of aromatic molecules; new developments.. Adv Cancer Res.

